# *Debaryomyces hansenii* strains differ in their production of flavor compounds in a cheese-surface model

**DOI:** 10.1002/mbo3.11

**Published:** 2012-06

**Authors:** Klaus Gori, Louise Marie Sørensen, Mikael Agerlin Petersen, Lene Jespersen, Nils Arneborg

**Affiliations:** 1 Department of Food Science, Food Microbiology, Faculty of Life Sciences, University of CopenhagenRolighedsvej 30, DK-1958 Frederiksberg C, Denmark; 2 Department of Food Science, Quality and Technology, Faculty of Life Sciences, University of CopenhagenRolighedsvej 30, DK-1958 Frederiksberg C, Denmark

**Keywords:** Cheese ripening, *Debaryomyces hansenii*, flavor production

## Abstract

Flavor production among 12 strains of *Debaryomyces hansenii* when grown on a simple cheese model mimicking a cheese surface was investigated by dynamic headspace sampling followed by gas chromatography-mass spectrometry. The present study confirmed that *D*. *hansenii* possess the ability to produce important cheese flavor compounds, primarily branched-chain aldehydes and alcohols, and thus important for the final cheese flavor. Quantification of representative aldehydes (2-Methylpropanal, 3-Methylbutanal) and alcohols (2-Methyl-1-propanol, 3-Methyl-1-butanol, and 3-Methyl-3-buten-1-ol) showed that the investigated *D*. *hansenii* strains varied significantly with respect to production of these flavor compounds. Contrary to the alcohols (2-Methyl-1-propanol, 3-Methyl-1-butanol, and 3-Methyl-3-buten-1-ol), the aldehydes (2-Methylpropanal, 3-Methylbutanal) were produced by the *D*. *hansenii* strains in concentrations higher than their sensory threshold values, and thus seemed more important than alcohols for cheese flavor. These results show that *D*. *hansenii* strains may have potential to be applied as cultures for increasing the nutty/malty flavor of cheese due to their production of aldehydes. However, due to large strain variations, production of flavor compounds has to be taken into consideration for selection of *D*. *hansenii* strains as starter cultures for cheese production.

## Introduction

The hemiascomycetes yeast *Debaryomyces hansenii* is a heterogeneous species able to grow in extreme environments, for example, high NaCl concentrations (Norkrans 1966; Gunde-Cimerman et al. 2009), which correlates with its importance for production of salty foods including different types of meat products and cheeses (Eliskases-Lechner and Ginzinger 1995; Jessen 1995; Gunde-Cimerman et al. 2009). In fact, *D*. *hansenii* is the dominant yeast species found on most cheese varieties, for example, surface-ripened cheeses (Reps 1993). During the initial ripening period, *D*. *hansenii* is favored on the cheese surface due to its ability to grow in the cheese environment with high NaCl concentrations, low pH, and lactate as the main carbon source. The growth of *D*. *hansenii* increases the pH of the cheese surface, and thus the growth of the less acid tolerant smear bacteria, by assimilation of lactate and production of alkaline metabolites such as ammonia (Petersen et al. 2002; Gori et al. 2007). Starter cultures of *D. hansenii* might be added even though they can be displaced by naturally occurring strains of *D*. *hansenii.* Studies of different types of cheeses have shown that the population of *D*. *hansenii* often develops from a heterogeneous to a homogenous population, sometimes only consisting of a single strain (Petersen et al. 2002). It has been assumed that *D*. *hansenii* originates from the brine, in which cheeses are salted in prior to ripening, or from the raw milk, because of survival of *D*. *hansenii* through pasteurisation (Seiler and Busse 1990; Mounier et al. 2006). However, investigation of the *D*. *hansenii* strains on the surface of Danish Danbo cheeses showed that the dominating *D*. *hansenii* strain neither originated from the raw milk, brine nor starter culture but from the dairy houseflora (Petersen et al. 2002).

Cheese flavor is one of the most important criteria determining consumer choice and acceptance (Hersleth et al. 2005; Caspia et al. 2006; Drake et al. 2008). Major cheese flavor compounds are sulfur compounds, ketones, aldehydes, esters, alcohols, lactones, and free fatty acids, which are produced from proteins, lipids, and lactose through numerous biochemical reactions (McSweeney and Sousa 2000; Marilley and Casey 2004; Ardö 2006). The general component balance theory states that cheese flavor is the outcome of a synergistic odor effect of the right blend of some of these compounds in a balanced proportion (Law 1984; Arora et al. 1995). None of the compounds represent cheese flavor by itself.

In addition to an indirect contribution of yeasts to the ripening process, new studies have shown that not only bacteria but also yeast may contribute to the ripening process in a direct way by the production of flavor compounds (Ferreira and Viljoen 2003; Das et al. 2005; De Wit et al. 2005; De Freitas et al. 2009). However, dairy-relevant yeast species contribute differently to the flavor production of the final cheeses. *Kluyveromyces lactis* and *Kluyveromyces marxianus* have been found to produce primarily esters (Martin et al. 2001; Leclercq-Perlat et al. 2004), whereas *Geotrichum candidum* have been found to produce primarily sulfur compounds (Martin et al. 2001; Spinnler et al. 2001). Also a few previous studies have focused on the flavor production of *D*. *hansenii* and found it primarily to produce aldehydes and alcohols (Martin et al. 2001; Arfi et al. 2002; Leclercq-Perlat et al. 2004; Arfi et al. 2005). However, to our knowledge, no studies have previously focused on the intraspecies variation with respect to production of these cheese flavor compounds within *D*. *hansenii* strains.

In the present study, we investigated the flavor compound production of 12 *D*. *hansenii* strains on a solid substrate mimicking a cheese surface using dynamic headspace sampling followed by gas chromatography-mass spectrometry (GC-MS) analysis.

## Materials and Methods

### Strains and propagation

The 12 *D. hansenii* strains used in the present study were all isolated from various stages of cheese production, that is, cheese, rennet, or brine (Table 1), and each of the strains were isolated from individual dairy sites. The *D*. *hansenii* strains were maintained at −80°C in yeast glucose peptone (YGP) broth (per liter: 5 g yeast extract [Difco Laboratories, Detroit, MI], 10 g glucose [Merck, Darmstadt, Germany], 5 g Bacto peptone [Difco Laboratories]) containing 20% (v/v) glycerol (Merck). The yeast strains were propagated in two steps. YGP (25 mL) was inoculated with 1 mL of freeze culture and incubated for 48 h at 25°C with shaking at 120 rpm. Cells were counted using a Thoma counting chamber, YGP (100 mL) was inoculated with 1 × 10^6^ cells per mL and incubated for 48 h at the same conditions as for the first propagation step.

**Table 1:** *Debaryomyces hansenii* strains included in the present study

Name	Origin	Source	References
CBS117	Rennet	Centraalbureau voor Schimmelcultures (CBS), Utrecht, The Netherlands	Tunblad-Johansson and Adler 1987; Petersen et al. 2001
CBS164	Cheese	Centraalbureau voor Schimmelcultures (CBS)	van den Tempel and Jakobsen 2000; Petersen et al. 2001
CBS766	Cheese	Centraalbureau voor Schimmelcultures (CBS)	Petersen et al. 2001; Groenewald et al. 2008; Jacques et al. 2009
CBS772	Rennet	Centraalbureau voor Schimmelcultures (CBS)	Petersen et al. 2001; Groenewald et al. 2008
CBS1800	Cheese	Centraalbureau voor Schimmelcultures (CBS)	Petersen et al. 2001; Klein et al. 2002; Petersen and Jespersen 2004
CBS8416	Brine	Centraalbureau voor Schimmelcultures (CBS)	Petersen et al. 2001; Petersen and Jespersen 2004
MD02	Cheese	Arla Innovation Center, Brabrand, Denmark	Petersen et al. 2002; Mortensen et al. 2005a; Gori et al. 2007
D18335	Cheese	University of Copenhagen, Faculty of Life Sciences, Frederiksberg, Denmark	Petersen et al. 2002; Mortensen et al. 2005a; Gori et al. 2007
BH24	Cheese	University of Copenhagen, Faculty of Life Sciences	van den Tempel and Jakobsen 1998
FB8	Cheese	University of Copenhagen, Faculty of Life Sciences	van den Tempel and Jakobsen 1998; Masoud and Jakobsen 2005
FB10	Cheese	University of Copenhagen, Faculty of Life Sciences	van den Tempel and Jakobsen 1998
FB12	Cheese	University of Copenhagen, Faculty of Life Sciences	van den Tempel and Jakobsen 1998

### Preparation of cheese agar

Unsalted, unripened Danbo cheese (200 g, 45% fat in dry matter) from a local dairy was added to 400 mL of auto-claved demineralized water and ground for 5 min using a hand-blender. Tri-sodium citrate-dihydrate (12.5 g) and NaCl (40 g) (both from Merck) were added, and the mixture was boiled for 1 h with magnetic stirring. In the meantime, Bacto agar (20 g) (Difco Laboratories) was added to 500 mL of demineralized water and autoclaved for 20 min at 121°C. After autoclaving, the temperature of the agar was adjusted to approximately 80°C, and the agar was added to the cheese suspension. The pH was adjusted to 5.0 with sterile-filtered 1 M HCl, and autoclaved mineralized water was added up to 1 L. The cheese agar was immediately poured into Petri dishes with 25 mL per dish and allowed to solidify at room temperature.

### Inoculation of cheese agar

Cheese agar was inoculated with 0.3 × 10^3^–1.8 × 10^3^ colony-forming units (CFU)/cm^2^ of each *D*. *hansenii* strain using a Drigalski spatula. The inoculated cheese agar plates were incubated for 12 days in perforated bags at 12°C corresponding to the initial cheese-ripening conditions for surface-ripened cheeses. A blank uninoculated cheese agar plate was incubated under the same conditions.

### Determination of growth and pH measurements

Yeast growth and pH were each measured for three individual cheese agar plates at the end of the incubation. For the determination of the CFU, whole cheese agar plates were mixed with saline peptone diluent (SPO) (0.1% [w/v] Bacto Peptone [Difco Laboratories], 0.85% [w/v] NaCl [Merck], 0.03% Na_2_H_2_PO_4_, 2H_2_O [Merck] adjusted to pH 5.6) in a stomacher bag to yield a 1:10 dilution. The mixture was homogenized using a Stomacher for 2 min at medium speed. From this dilution, 10-fold dilutions were prepared in SPO, which were spread-plated in duplicates on YGP agar. Plates were incubated at 25°C for three to five days. Measurements of pH were performed with a surface electrode (Inlab 426, Mettler-Toledo, Glostrup, Denmark) connected to a pH meter (1120, Mettler-Toledo). Calibration of the electrode was performed in buffers with pH 4.01 and 7.00 (Radiometer, Brønshøj, Denmark).

### Determination of volatile compound production

Volatile compounds were measured in triplicate at the end of the incubation using dynamic headspace sampling followed by GC-MS analysis. Each headspace sample consisted of the content from two cheese agar plates (40–45 g). A slurry was made by addition of 65 mL of phosphate buffer (per liter: 50 g K_2_HPO_4_, 10 g KH_2_PO_4_, and 100 g NaCl [pH 6.8]) and 0.5 mL of 4-Methyl-1-pentanol (50 mg/L) as internal/positive control standard that was homogenized with an Ultra-Turrax T25 (Janke & Kunkel, Staufen, Germany) at 13.500 rpm for 1.5 min. The slurry was equilibrated to 30°C in a water bath and then purged with nitrogen (100 mL min^−1^) for 45 min with stirring at 200 rpm. Volatile compounds were collected on Tenax-TA traps (250 mg, mesh size 60/80, density 0.37 g mL^−1^, Buchem bv, Apeldoorn, The Netherlands) at 30 ± 1°C. The trapped volatiles were desorbed using an automatic thermal desorption unit (ATD 400, Perkin Elmer, Norwalk, CT) and automatically transferred to a gas chromatograph-mass spectrometer (GC-MS, G1800A, GCD System, Hewlett-Packard, Palo Alto, CA). Separation of volatiles was carried out on a DB-Wax capillary column (J&W Scientific, 30 m long × 0.25 mm internal diameter, 0.25 μm film thickness). The mass spectra were recorded in electronic impact mode at 70 eV in a mass/charge ratio from 15 to 300 m/z. Data analysis was performed in the software program MSDchemstation (Version E.01.00.237, Agilent Technologies, Santa Clara, CA). Identification of compounds was based on comparison with a mass spectral database (Wiley275.L, HP product nr G1035A). One characteristic quantifier ion and two-three qualifier ions were selected for each compound. The compounds were detected when the quantifier and qualifier ions were detected with less than 35% deviation from the expected ratio (Table 2) and the peak area of the quantifier ion was used for quantification. Calibration curves based on matrix-spiked samples in triplicates were performed for the aldehydes (2-Methylpropanal, 3-Methylbutanal) and the alcohols (2-Methyl-1-propanol, 3-Methyl-1-butanol, and 3-Methyl-3-buten-1-ol). The compounds were spiked in to the cheese agar in concentrations 125, 250, and 500 ppb. Concentrations of flavor compounds were corrected using the estimated concentration in the matrix (*B_Y_*_= 0_).

**Table 2:** Parameters for quantification. Retention time (Rt), quantifier ion (Quan), and qualifier ions (Qual) with relative abundance to Quan in parenthesis. Slope, intercept, and correlation coefficient for matrix-spiked calibration curves and estimated concentration in matrix (*B_Y_*_= 0_) used for correction

				Calibration curves			
				
Volatile compound	Rt (min)	Quan (m/z)	Qual (m/z) (%)	Slope	Intercept	*R*^2^	*B_Y_*_= 0_
2-Methylpropanal	2.636	72	41 (127), 27 (52), 29 (40)	42,300	−140,800	0.9939	0
3-Methylbutanal	3.536	58	57 (41), 71 (47), 86 (12)	84,800	−194,100	0.9896	0
2-Methyl-1-propanol	8.506	43	41 (70), 42 (60), 31 (37)	31,000	1,000,000	0.9335	32
3-Methyl-1-butanol	13.997	55	70 (71), 39 (23)	92,600	4,000,000	0.9417	43
3-Methyl-3-buten-1-ol	15.129	56	41 (92), 68 (90), 86 (32)	29,600	505,700	0.9096	17

### Statistical analysis

To test whether there was a significant difference (95% confidence level) between the cheese samples a one-way ANOVA using Tukey's Honestly Significant Difference (HSD) test was performed with JMP 8 (SAS Institute, Cary, NC).

## Results and Discussion

### Growth and acidification capacity of *D. hansenii* strains

The 12 *D*. *hansenii* strains reached between 1.6 × 10^7^ and 8.7 × 10^7^ CFU/cm^2^ when grown on cheese agar added 4% (w/v) NaCl for 12 days at 12°C (Table 2). The yeast count on surface-ripened cheese has been found to vary from below 100 CFU/cm^2^ up to 10^8^ CFU/cm^2^ depending on cheese type and whether yeast culture is added (Eliskases-Lechner and Ginzinger 1995). The counts of *D*. *hansenii* found in the present study were similar to what has been found previously on Danish surface-ripened cheeses (Petersen et al. 2002).

The *D*. *hansenii* strains increased pH from 5.2 to between 7.7 and 8.2 except one strain (CBS 164), which only increased the pH to 6.2, and thus apparently had a lower de-acidifying capacity (Table 3). Generally, the pH of the cheese surface has to be 5.8 or even higher before growth of smear bacteria is observed (Corsetti et al. 2001).

**Table 3:** Growth (colony-forming units per cm^2^) and pH changes in cheese agar inoculated with the indicated *D. hansenii* strains

*Debaryomyces hansenii* strain	Growth^1^,^2^,^3^ (CFU/cm^2^) after 12 days	pH^3^ after 12 days
Blank	-	5.2 ± 0.1^H^
CBS117	2.0 × 10^7^ ± 0.4 × 10^7EF^	8.0 ± 0.0^CD^
CBS164	5.7 × 10^7^ ± 1.1 × 10^7B^	6.2 ± 0.1^G^
CBS766	3.2 × 10^7^ ± 0.2 × 10^7D^	8.2 ± 0.1^A^
CBS772	8.6 × 10^7^ ± 0.7 × 10^7A^	7.8 ± 0.1^EF^
CBS1800	3.7 × 10^7^ ± 0.7 × 10^7CD^	8.2 ± 0.1^A^
CBS8416	1.6 × 10^7^ ± 0.1 × 10^7F^	8.1 ± 0.1^ABC^
MD02	5.5 × 10^7^ ± 0.9 × 10^7BC^	8.2 ± 0.1^AB^
D18335	3.4 × 10^7^ ± 0.7 × 10^7D^	7.8 ± 0.1^EF^
BH24	3.4 × 10^7^ ± 0.4 × 10^7D^	8.0 ± 0.0^BCD^
FB8	3.3 × 10^7^ ± 0.2 × 10^7D^	7.9 ± 0.0^DE^
FB10	2.8 × 10^7^ ± 0.6 × 10^7DE^	7.7 ± 0.1^F^
FB12	2.5 × 10^7^ ± 0.2 × 10^7DE^	7.9 ± 0.0^DE^

1 The cheese agar was added with 4% NaCl in water (w/w) and incubation was at 12°C for 12 days. The initial inocula were 0.3 × 10^3^–1.8 × 10^3^ CFU/cm^2^.

2 Average values (*n* = 3) with RSD <21%.

3 Values in same column not marked by same superscript capitals are significantly different using one-way ANOVA with Tukey's HSD test (≥95% confidence).

### Production of volatile compounds by *D. hansenii* strains

Due to the prevalence of *D. hansenii* on cheese surfaces, *D. hansenii* strains are obvious candidates as starter cultures for cheese ripening (Bockelmann 2002). Starter cultures of *D. hansenii* should at least process the ability to grow at low pH, low temperature, and high NaCl concentrations, which promote the growth of *D. hansenii* in the initial period of ripening and thus acidification of the cheese surface. However, additional technological properties including cheese flavor production may be of importance. Concerning the cheese flavor production, it is noteworthy that cheese flavor production is not solely due to one microorganism but a mixture of various microorganisms, and thus cheese ripening is highly dependent on microbial interactions. Several studies have shown that ripening cultures do not necessarily establish well in the cheese-making environment and are outcompeted by the indigenous flora in the dairy (Petersen et al. 2002; Mounier et al. 2005). However, the aim of the present study has been to perform an initial screening for flavor production among *D. hansenii* strains in a cheese-surface model.

Dynamic headspace extraction followed by GC-MS has been shown suitable for detection and quantification of volatile flavor compounds produced by dairy-relevant yeast species (Berger et al. 1999; Martin et al. 2001; Leclercq-Perlat et al. 2004; Arfi et al. 2005; Sørensen et al. 2011). In the present study, we used dynamic headspace extraction followed by GC-MS to investigate the production of flavor compounds of 12 strains of *D*. *hansenii* due to its high prevalence and importance during cheese ripening. All 12 strains primarily produced aldehydes (2-Methylpropanal, 2-Methylbutanal, and 3-Methylbutanal) and alcohols (2-Methyl-1-propanol, 2-Methyl-1-butanol, 3-Methylbutan-1-ol, and 3-Methyl-3-buten-1-ol) in higher levels than the blank sample (un-inoculated cheese agar) (results not shown). This confirms findings from previous studies that *D*. *hansenii* strains primarily produce aldehydes and alcohols as cheese flavor compounds (Martin et al. 2001; Arfi et al. 2002; Leclercq-Perlat et al. 2004; Arfi et al. 2005; Sørensen et al. 2011).

**Figure 1:**
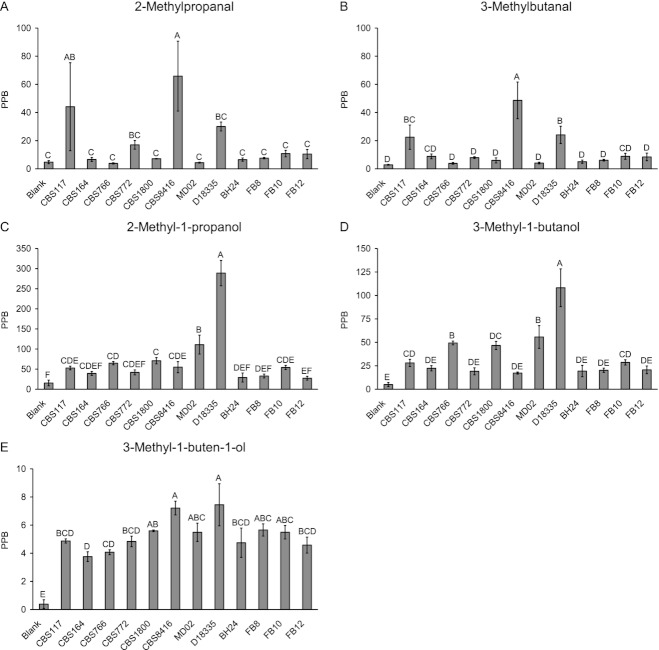
Contents of branched-chain aldehydes and alcohols in cheese agar inoculated with the indicated *D*. *hansenii* strains. (A) 2-Methylpropanal, (B) 3-Methylbutanal, (C) 2-Methyl-1-propanol, (D) 3-Methyl-1-butanol, and (E) 3-Methyl-1-buten-1-ol. Columns not marked by same superscript capitals have significantly different average values (*n* = 3) using one-way ANOVA with Tukey's HSD test (≥95% confidence).

Representative aldehydes (2-Methylpropanal, 3-Methyl-butanal) and alcohols (2-Methyl-1-propanol, 3-Methyl-1-butanol, and 3-Methyl-3-buten-1-ol) produced by the 12 *D*. *hansenii* strains were quantified by matrix-spiked calibration curves (Table 2). Quantitatively, great variation was observed in the production of these compounds among the investigated *D*. *hansenii* strains, as the levels varied more than 10-fold for both the aldehydes and alcohols (Fig. 1). The aldehydes, 2-Methylpropanal and 3-Methylbutanal, have been shown to be decisive for the nutty flavor of cheddar cheese (Avsar et al. 2004) as well as important for the flavor of parmesan cheese (Qian and Reineccius 2003) and thus likely important cheese flavor compounds of surface-ripened cheeses. In the present study, only three *D*. *hansenii* strains (CBS117, CBS8416, and D18335) produced significant levels of 2-Methylpropanal and 3-Methylbutanal. These levels were within or higher than the sensory threshold values reported to be in the range of 0.4–43 ppb for 2-Methylpropanal and 5-13 ppb for 3-Methylbutanal (Curioni and Bosset 2002; Burdock et al. 2005). However, the levels were much lower than the levels between 500 and 700 ppb previously found in cheese (Qian and Reineccius 2003).

2-Methylpropanal and 3-Methylbutanal are branched-chain aldehydes derived from catabolism of the amino acids valine and leucine, respectively (Yvon and Rijnen 2001; Ardö 2006). The most common biosynthetic pathway starts by removal of the amino group by the act of the amino-transferase resulting α-keto acids, which are decarboxylated to aldehydes. However, these aldehydes can in turn be reduced to alcohols, which have been described to have flavors as fruity, solvent, alcoholic, fusel, and pomace (Curioni and Bosset 2002; Burdock et al. 2005). In the present study, more than half of the investigated *D*. *hansenii* strains produced significantly higher levels of the alcohols 2-Methyl-1-propanol and 3-Methyl-1-butanol corresponding to reduction of the aldehydes 2-Methylpropanal and 3-Methylbutanal. These levels were, however, much lower than the sensory threshold values reported to be in the range of 360–3300 ppb for 2-Methyl-1-propanol and 250-4100 ppb for 3-Methyl-1-butanol (Curioni and Bosset 2002; Burdock et al. 2005). In addition, the level of 3-Methyl-1-butanol was lower than the level of 190 ppb previously found in cheese (Qian and Reineccius 2003). Finally, all the investigated *D*. *hansenii* strains produced significant levels of the alcohol 3-Methyl-3-buten-1-ol. To our knowledge, no information of the role of 3-Methyl-3-buten-1-ol on cheese flavor has been reported.

The *D. hansenii* species has previously been found to be genetically heterogeneous, resulting in different technological properties among strains (Petersen and Jespersen 2004). Thus, genetic differences could similarly explain the variations in flavor production among strains found in the present study. However, the six *D. hansenii* strains obtained from the CBS strain collection belong to five different genotypic clusters, varying in numbers and sizes of chromosomes (Petersen and Jespersen 2004), and their flavor production is virtually similar (Fig. 1). These results indicate that no clear-cut correlation seems to exist between the geno- and phenotype of *D. hansenii* when considering flavor production. Instead, the great variation with respect to production of flavor compounds among the *D*. *hansenii* strains may be a result of different proteolytic activity and ability to degrade peptides to the precursor amino acids, different activity of amino-transferases or α-keto acid decarboxylases, different activity of alcohol dehydrogenases and aldehyde oxidases or finally un-resolved result of time-course production. However, further investigations have to be done on this issue by, for example, proteome analysis and relevant enzyme activity assays.

Interestingly, the strain *D*. *hansenii* D18335, which in the present study was found to produce both high amounts of aldehydes and alcohols, has been shown to fully dominate the surface of Danish Danbo cheese after three days of ripening (Petersen et al. 2002). The origin of *D*. *hansenii* D18335 was found to be the houseflora. The dominance of *D*. *hansenii* D18335 has previously been shown to be due to a better adaption to the environmental stress conditions present during the production of surface-ripened cheeses resulting in particular abilities with respect to adhesion, NaCl tolerance, and lactate assimilation (Petersen et al. 2002; Gori et al. 2005; Mortensen et al. 2005a); adhesion being the prerequisite for growth, deacidification, and production of potential flavor compounds on the solid cheese surface. The present study shows that *D*. *hansenii* D18335 also may have particular abilities with respect to the production of important flavor compounds. Thus, this strain may have a potential as starter culture for the production of surface-ripened cheeses.

In conclusion, *D*. *hansenii* may have potential to be applied as starter cultures for contribution to the final cheese flavor by their production of branched-chain aldehydes primarily responsible for nutty/malty flavor notes. However, strain variation in production of cheese flavor compounds was significant indicating that strains of *D*. *hansenii* are heterogeneous in their contribution to cheese flavor production and suggesting that the production of cheese flavor compounds is taken into consideration for selection of *D*. *hansenii* strains as starter cultures for cheese production.
